# A very unusual anatomical variation and complication of common iliac artery and ureter in retroperitonoscopic ureterolithotomy

**DOI:** 10.4103/0972-9941.78348

**Published:** 2011

**Authors:** Emre Huri, Turgay Akgül, Tolga Karakan, Mustafa Sargon, Cankon Germiyanoğlu

**Affiliations:** Department of Second Urology Clinic, Ankara Training and Research Hospital, Turkey; 1 Department of Anatomy, Hacettepe University, Turkey

**Keywords:** Aneurysm, common iliac artery, ureter

## Abstract

Anatomical localization of theureter comes along psoas major muscle and crosses over common iliac artery bifurcation. Common iliac artery aneurysm and impacted atherosclerosis are a rare condition that should be differed from the impacted ureter stone to avoid from undesirable complication. In this case, we present a very unusual anatomical variation and complication of common iliac artery and ureter in retroperitonoscopic ureterolithotomy.

## INTRODUCTION

Retroperitonoscopic ureterolithotomy is an effective procedure to manage the large and impacted ureteral stones with minimal complication. However, in this kind of surgery, the anatomical relations of the ureter and the adjacent organs should be well-known by surgeon. The prevalence of isolated aneurysms of iliac artery in general population is very rare and was estimated to be 0.03% according to an autopsy study.[1] The common iliac artery is affected in 70% of cases, the internal iliac artery in 20% of cases and the external iliac artery in 10% of cases.[2] In this case, we present a possible complication in retroperitonoscopic ureterolithotomy procedure that is caused by an unusual anatomical variation of the common iliac artery, coexistence of atherosclerosis and aneurism, mimicking the ureteral stone. However, the purpose of the manuscript is to describe the surgical problems caused by an anatomical variation of the iliac artery.

## CASE REPORT

**Figure 1:**
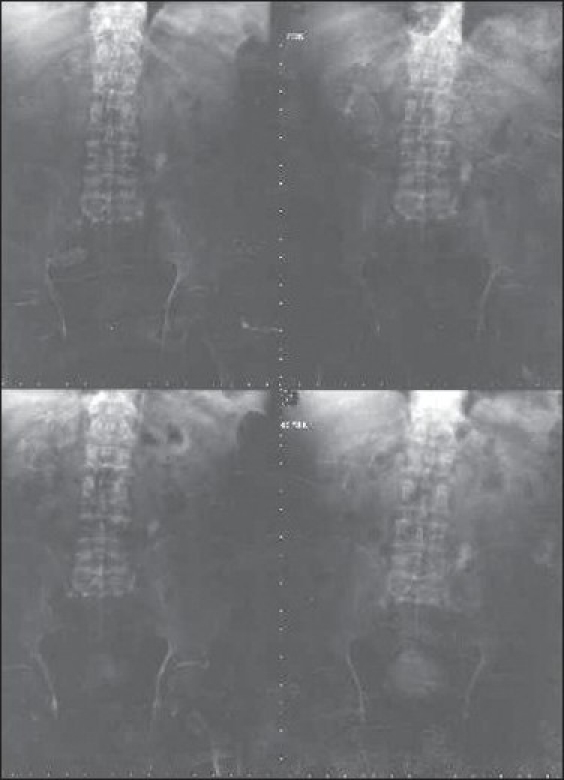
IVU pictures of ureteral stone without any suspicion of calcifi ed bulging.

**Figure 2:**
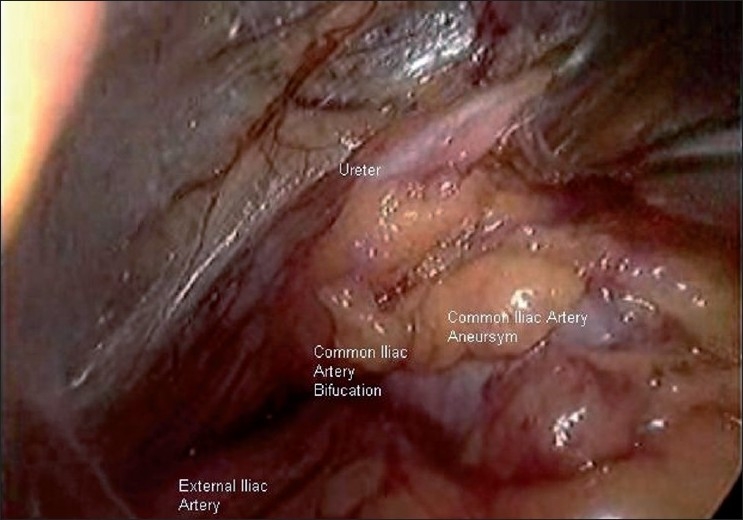
The view of common iliac artery aneurysm and related anatomical structures in laparoscopic vision.

**Figure 3:**
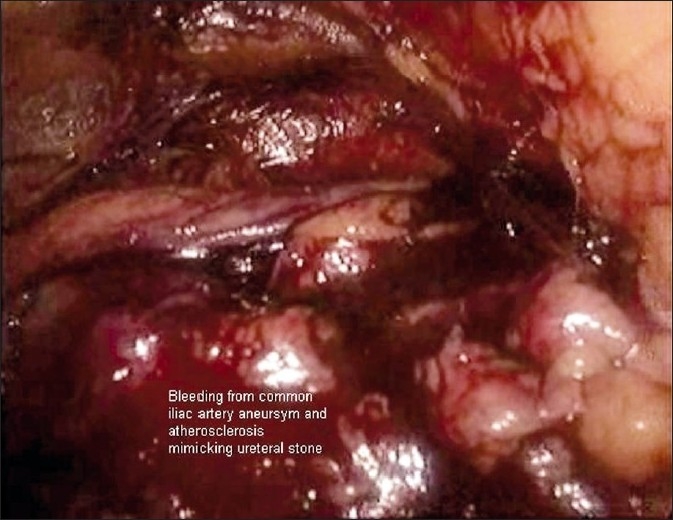
The unusual complication bleeding from common iliac artery aneurysm mimicking the ureter and the impacted ureter stone.

A 55-year-old male patient suffering from left loin pain was admitted to the outpatient clinic. Following the required urological evaluation a left ureteral stone measuring 22×30 mm was detected. Non-enhanced, stone protocol, abdominopelvic spiral computed tomography (CT) was performed to evaluate the stone location and other anatomical structures. Plain X-ray KUB and IVU pictures were confirmed the left ureter stone [Figure 1]. Retroperitonoscopic ureterolithotomy was planned. The proper dissection was performed retroperitonocopically. Psoas major muscle, genitofemoral nerve, lower pole of left kidney, ureter were identified and dissected. At the level of mid-upper ureter, under the ureter, a nearly 2×2 cm bulging was observed and thought to be an anatomical variation of the ureter. The tactile sense of the bulging surface was hard and smooth. Therefore, a gentle aspiration was tried but not achieved. Mini-incision was performed with endoscalpel and there was sudden bleeding. Then, we converted the procedure to an open surgery because of an unexpected and uncontrolled bleeding. During open exploration, a common iliac artery aneurism and atherosclerotic structures were detected at the same level that was mimicking the ureter and stone. The incision on the aneurism was closed with 6/0 polyglicolic acid. The common iliac artery aneurism was located at the level of bifurcation and ureteral cross-side [Figure 2]. Aneurysmatic dilatation and calcified atherosclerosis of common iliac artery and external iliac artery mimicked the ureteral stone and caused the unusual complication as well [Figure 3]. The patient was discharged at the fourth day without postoperative complication.

## DISCUSSION

In exceptional cases, common iliac artery aneurism is presented as a urinary obstructive factor that leads to hydronephrosis in the literature.[3] In these cases, a CT scan demonstratesan encased ureter and aneurysm of common iliac artery. However, in our policy, to evaluate the urinary stones, routine CT scan is not obligatory if IVU gives proper findings about the stone. Mieog *et al*, showed the inflammatory aneurysm of the common iliac artery mimicking appendicitis.[4] However, Dittrick *et al* reported a case of an idiopathic-calcified infrarenal aortic aneurysm in a child with a non-specific lymphadenopathy.[5] Although there is high level of anatomical knowledge, it is still possible to mislead the anatomical variation and thereby lead to complication. Because of the anatomical relation of common iliac artery with ureter, aneurisms and atherosclerosis of common iliac artery or branches should be in mind while doing the dissection to the ureter.

Laparoscopic surgery is recommended for impacted and large ureteral stone. In this case, although the proper dissection was applied along to the ureter, the suspicious anatomical variation of common iliac artery mimicking the ureter stone was the main reason to convert the session to an open surgery. The non-enhanced CT scan was preferred to view the ureter stone, so that the vascular aneurism could not be detected with this radiological investigation.

In conclusion, the anatomical variation of common iliac artery may lead to the complication during laparoscopic ureterolithotomy even if the routine preoperative radiological evaluation that focused on the ureter stone, is performed.

## References

[1] Zimmermann A, Kuehnl A, Stefan S, Eckstein HH (2009). Idiopathic aneurysm of the common iliac artery in a 11-year old child. J Vasc Surg.

[2] Krupski WC, Selzman CH, Floridia R, Strecker PK, Nehler MR, Whitehill TA (1998). Contemporary management of isolated iliac aneurysms. J Vasc Surg.

[3] Maeda S, Ogura K, Arai Y, Takeuchi H, Yoshida O, Mori K (1993). Ureteral obstruction caused by aneurysm of iliac artery. Hinyokika Kiyo.

[4] Mieog JS, Stoot JH, Bosch JJ, Koning OH, Hamming JF (2008). Inflammatory aneurysm of the common iliac artery mimicking appendicitis. Vascular.

[5] Dittrick K, Allmendinger N, Wolpert L, Windels M, Drezner D, Lapuck S (2002). Calcified abdominal aortic aneurysm in a 12-year-old boy. J Pediatr Surg.

